# Effects of the Community Score Card approach on reproductive health service-related outcomes in Malawi

**DOI:** 10.1371/journal.pone.0232868

**Published:** 2020-05-19

**Authors:** Sara Gullo, Christine Galavotti, Anne Sebert Kuhlmann, Thumbiko Msiska, Phil Hastings, C. Nathan Marti

**Affiliations:** 1 CARE USA, Atlanta, Georgia, United States of America; 2 College for Public Health & Social Justice, Saint Louis University, St. Louis, Missouri, United States of America; 3 CARE Malawi, Lilongwe, Malawi; 4 Far Harbor, LLC, Austin, TX, United States of America; Ravensburg-Weingarten University of Applied Sciences, GERMANY

## Abstract

**Background:**

Social accountability approaches are increasingly being employed in low-resource settings to improve government services. In line with the continuous quality improvement (CQI) philosophy that quality is the product of a linked chain, collaborative social accountability approaches like the Community Score Card (CSC) aim to empower clients *and* frontline service providers to transform their own lives and hold public officials to account for state obligations. Despite being a critical focus of collaborative social accountability approaches, to our knowledge, a quantitative survey of health workers to understand the impact of these approaches on their self-reported responsibilities and service provision has not been conducted. To fill this gap, we carried out a quantitative survey with health workers to assess the CSC’s impact on health worker-reported service responsibilities and provision and complement women’s self-reports.

**Methods:**

We evaluated the effect of the CSC on reproductive health-related outcomes using a cluster-randomized design in Ntcheu district, Malawi. We matched 10 pairs of health facilities and surrounding catchment communities; one from each pair was randomly assigned to the intervention and control arms. The intervention communities and health workers each completed 3–4 cycles of the CSC process by endline. We then surveyed all health workers in the 20 intervention and comparison sites at endline (n = 412) to estimate the intervention’s impact.

**Results:**

Significantly (p < .05) more health workers in the CSC intervention areas compared to control areas reported responsibility for antenatal care, comprehensive antenatal care counseling, recording of the number of pregnant and postpartum women seen each month, and the average age of their last family planning client was younger. In addition, marginally significantly (p < .10) more health workers in treatment versus control areas report visiting women at their home at least once during their pregnancy. However, health worker-reported responsibility for HIV testing was significantly lower in intervention areas than in control.

**Conclusions:**

The CSC aims to empower health workers to collaborate with the community and rest of the health system to identify and overcome the diverse and context-specific range of performance barriers they face. In doing so, it aims to support them to demand and ensure quality care for themselves from the health system so they can, in turn, deliver quality services to clients. Our results contribute to the evidence that the CSC may hold promise at improving service provision. While there is increasing evidence that collaborative social accountability approaches like the CSC are effective means to improving reproductive health-related service provision and outcomes in low-resource settings, additional research is needed.

## Introduction

Many healthcare workers in developing countries face challenging conditions, including inadequate and delayed salaries, competency gaps, heavy workloads, ambiguous responsibilities, lack of opportunities for growth, poor living and difficult working conditions, lack of autonomy, barriers to inter-professional collaboration and poor treatment by colleagues and patients [1,2,3,4]. These conditions not only result in burnout, stress, and poor retention for providers, but they also result in poorer quality care [1,3]. The connection between poor working conditions for providers and providers in turn providing poor quality of care to clients is in line with continuous quality improvement (CQI) philosophy. This philosophy suggests quality is a product of a linked chain in which each person is a customer of the people in the process preceding theirs and posits that for an organization to function well and provide quality care to its external customers it has to take care of the quality needs of its workers or internal customers [4,5,6,7]. A study from Ghana exploring the quality chain found that health workers perceived they do not receive ‘people-centered care’ from their employers, despite being asked to provide it to clients (i.e., the values they are being asked to hold for external clients are not being held for them by the health system). This hypocrisy weakened the message from the health system and in some cases lead to health worker apathy and less motivation to respond to external clients’ needs [7].

Social accountability is understood as “ongoing and collective effort to hold public officials to account for the provision of public goods which are existing state obligations” [8], including the delivery of quality services. While some social accountability approaches are adversarial others take a collaborative approach [9], recognizing that in many cases frontline health workers in developing countries are also disempowered and face challenging conditions. Collaborative social accountability approaches that improve dialogue between health workers and communities can lead to alliances to negotiate with higher level authorities for improvements [10]. Interestingly, a review of provider responsiveness to social accountability initiatives found health provider receptivity to citizen demands is mediated by the extent to which the social accountability initiatives provide personal and professional support to providers [11]. In addition, a review of evidence on interventions promoting external participation and accountability in low- and middle-income countries suggests that collaborative approaches to service provider engagement in transparency and accountability processes appear more effective than confrontational approaches and that many such interventions experience challenges stemming from lack of positive engagement with supply-side actors [12]. The collaborative approach to social accountability is line with the CQI philosophy that quality is a product of linked chain.

One such collaborative social accountability approach, CARE’s Community Score Card (CSC), aims to build “collaborative capacity” between service users and providers, empowering community members *and* service providers to identify obstacles and solutions in efforts to improve service utilization and quality [13]. Within the health sector, the CSC especially targets the frontline health workers who are the backbone of the health system but often feel disengaged and powerless in the decision-making process. Furthermore, the CSC involves district-level government officials and other power holders to help spur institutional and systemic change that can improve the working environment for health workers. By taking this approach, the CSC aims to improve two links in the quality chain (i.e., the external client and frontline provider links) and strengthen the relationship between the two. This is unlike many social accountability approaches that focus only on the external link in the quality chain.

In the health sector, there is promising evidence that collaborative social accountability approaches—like the CSC and the similar Citizen Voice and Action (CVA) approach—can improve relationships and spaces for negotiation between the community and health providers, and improve community empowerment, provider responsiveness, and ultimately enhance the availability, access, quality and uptake of services [12,13,14,15,16,17,18,19,20].

While it is important to understand how collaborative social accountability approaches impact client-reported health behaviors and service delivery experiences, to fully grasp the impact of these approaches it is critical to understand how these approaches impact health worker-reported service provision-related behaviors and experiences. This is important not only because frontline health workers play a critical role in the functioning of these social accountability approaches, but also because these approaches may lead to changes in service delivery and health worker behaviors that can be best (and is some cases only) ascertained from health worker self-reports. While several studies examining health sector collaborative social accountability approaches have used community and client-reported qualitative and quantitative data as well as process data (e.g., Score Card data) to understand the impact of these approaches, there has been less published on the impact of these approaches using health worker-reported service provision-related data [12,13,14,15,16,17,18,19,20]. To our knowledge, a quantitative survey of health workers to better understand a collaborative social accountability approach’s impact on their self-reported responsibilities and service provision as well as governance-related outcomes has not been carried out.

This paper presents the findings from a quantitative survey with health workers, exploring the CSC’s impact on health worker responsibilities and service provision in Malawi. This evidence is part of a cluster-randomized controlled evaluation examining the effect of the CSC on reproductive health-related outcomes in Malawi. In addition to the health worker survey, the evaluation also included Score Card data as well as a quantitative survey with women [17,19]. The health worker survey was used to bring the health workers’ voice to the evaluation of the CSC, which is often missing from evaluations of collaborative social accountability interventions. We sought to bring this voice through rigorous data collection, just as was done with the clients, and to triangulate this data with the women’s survey and Score Card data. The women’s survey showed that the CSC intervention had the following effect: significantly increased community health worker (CHW) visits to women during pregnancy and the postnatal period and women’s satisfaction with services, compared with control areas; had a significant effect on the use of modern contraception, with an estimated 57% greater use in the intervention versus control condition at endline; and improved spaces for negotiation and dialogue between women and health workers [17,19]. Analysis of the Score Card data, which included both community- and provider-rated indicators on areas prioritized to drive improvement efforts, showed that all 13 indicators (e.g., relationship between providers and communities, commitment of service providers, and level of youth and male involvement in reproductive health issues) improved from first to last scoring, with several improving significantly [17]. Complementing these findings, here we will present results from the health worker survey, sharing the CSC impact story using frontline health worker self-reported data.

## Methods

### Study setting

Much of Malawi’s population still lives in rural areas and relies on subsistence agriculture. The country’s health indicators remain relatively poor despite recent improvements. Ntcheu district lies near the border with Mozambique about half-way along the road between the country’s biggest cities of Lilongwe and Blantyre. Ntcheu has three hospitals and 33 health facilities administered by either the Malawi Ministry of Health (MOH) or the Christian Health Association of Malawi (CHAM). In 2011, 26 health facilities offered Prevention of Mother to Child Transmission of HIV (PMTCT) services, 6 provided basic emergency obstetric care (bEmOC), and 22 offered youth-friendly services. Family planning services were offered at all the MOH health facilities and some CHAM facilities.

### Intervention theory of change and description

**Fig 1:**
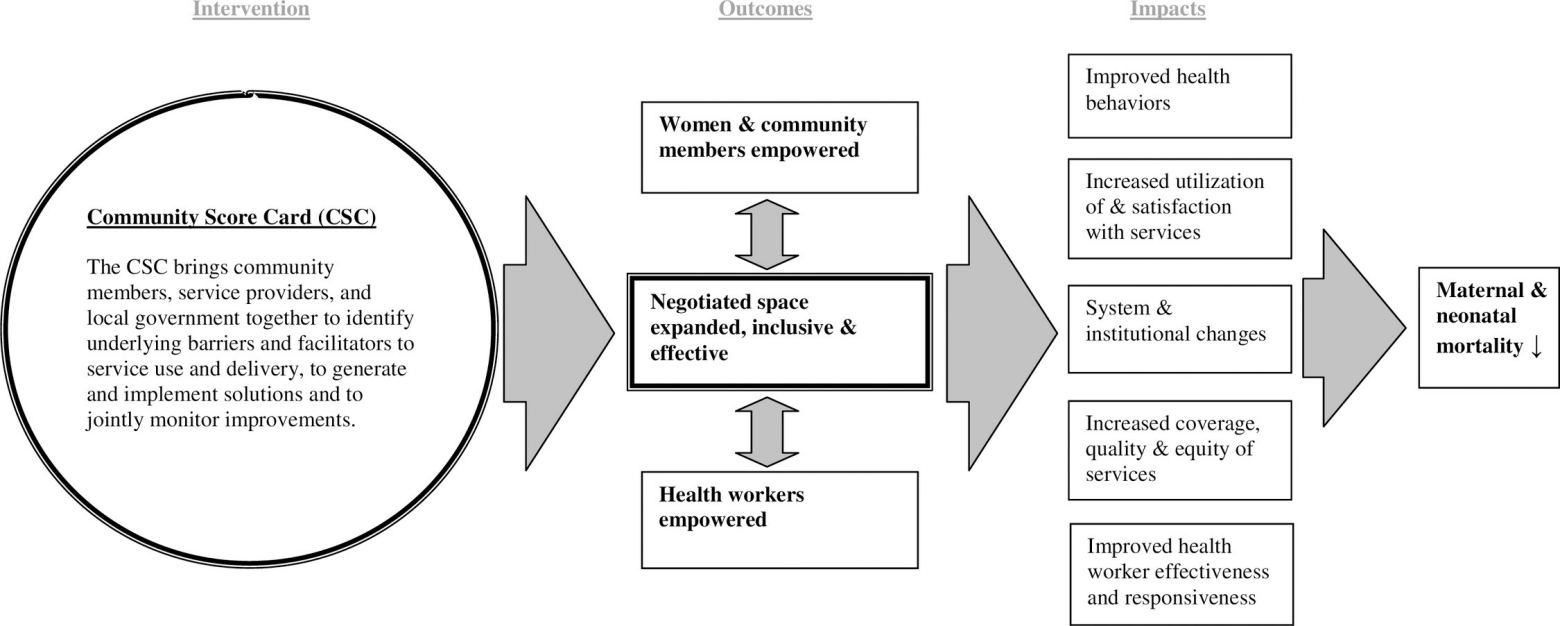
CARE’s Community Score Card theory of change.

The theory of change underlying the CSC intervention (Fig 1) suggests that bringing together community members, health workers, and local officials to a) identify barriers and facilitators of service use and delivery, b) develop and implement local solutions to overcome these barriers, and c) jointly monitor improvements will result in governance outcomes. These governance outcomes include empowered women and communities, empowered health workers, and new and expanded spaces for inclusive, effective dialogue and negotiation between the two. These governance outcomes interact and lead to a) improved health behaviors, increased service utilization and satisfaction with services among users, b) systems and institutional changes, and c) higher quality and more equitable service delivery by providers. Ultimately, these changes should decrease maternal and neonatal mortality in communities. As the theory of change hinges on equal participation and actions from both the community-side and health-delivery-side, women’s and health workers’ quantitative surveys as well as process data were carried out to examine the theory of change. The results from the women’s survey and process data have been published [17,19], and this paper presents the health worker survey service delivery-related results.

**Fig 2:**
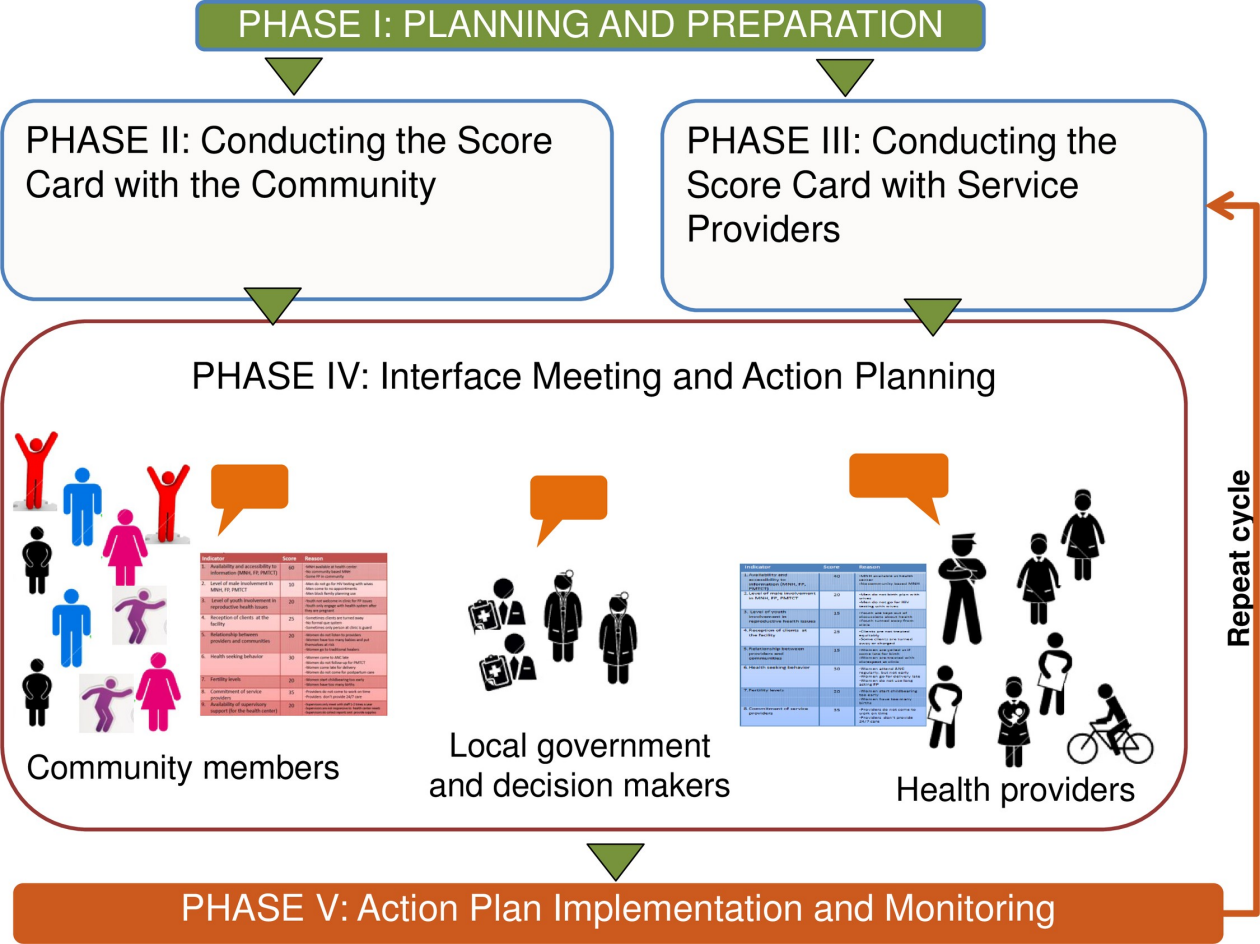
CARE’s Community Score Card process (5 phases).

The CSC intervention consists of five phases (Fig 2). The first phase of the CSC intervention involves planning and preparation. This stage involves identifying the sectoral and geographic scope of the initiative, understanding the context and barriers both service providers and users face, training facilitators, and securing cooperation and buy-in from all participating parties, including government officials as they are key partners in facilitating and responding to issues surfaced through the process. In Phase 2, issue generation, focus group discussions are carried out with community members (separated into groups such as men, women, youth, etc.) to identify and prioritize issues they are facing in accessing and utilizing services. Identified issues are organized into themes and a measurable indicator is developed for each theme (e.g., the relationship between providers and the community). The indicators are then verified and scored from 0 to 100 by the community, generating a Score Card. The community also indicates reasons for why a particular score was given and creates suggestions for improvement. The same process of issue generation and indicator development is conducted with service providers in Phase 3; through focus group discussions, service providers identify issues they are facing in delivering quality services, develop and score indicators, give reasons for the scores, and make suggestions for improvement. Phase 3 can occur either after or concurrently with Phase 2. Phase 3 is critical as it ensures that providers, who also often face significant challenges and are often disempowered have an opportunity to surface what is preventing them from delivering quality services. During Phase 4, the interface meeting, community members, service providers, local government officials and other power holders come together to discuss the community and service provider Score Cards, issues and priorities. This dialogue gives way to locally identified solutions to overcome barriers to service utilization and provision and an action plan for improvement. The action plan consists of items that need to be carried out by the community, service providers, local government and other power holders to improve service utilization and quality. Finally, Phase 5 involves action plan implementation, monitoring, and evaluation in which community members, service providers, government staff and additional power holders all have a role to play in implementing solutions as well as reviewing and monitoring progress on indicators. This cycle is repeated (minus the initial planning and preparation stage) every six months: communities and service providers reconvene to discuss issues (and generate new ones, if needed), re-score the indicators and discuss reasons for changes, and then meet in an interface meeting to review their respective Score Cards, in an on-going cycle of problem identification, solution generation, implementation of improvements, and mutual accountability. This process aims to foster conditions to build stronger relationships between community members and providers. It also aims to shift power to community members and frontline service providers so they can become self-learning, self-adaptive and self-leading in the co-creation of health and well-being.

The CSC intervention evaluated here focused on maternal and reproductive health-related outcomes such as family planning (FP), antenatal care (ANC) and postpartum care service utilization, and use of the health facility for labor and delivery. CARE Malawi in partnership with the local government District Health Management Team (DHMT) facilitated the CSC processes with service providers, including both facility-based service providers and CHWs, such as Health Surveillance Assistants (HSAs), and community members in the 10 intervention sites. All of the health facilities and workers that participated in the process were supported by the government of Malawi and administered either by the MOH or CHAM. Half of the intervention sites completed 4 cycles of the CSC process by the start of the endline data collection while the other half had completed 3 cycles. The Score Card indicators and results from the Score Card analysis are presented in Gullo et. al 2017 [17].

### Study design

**Fig 3:**
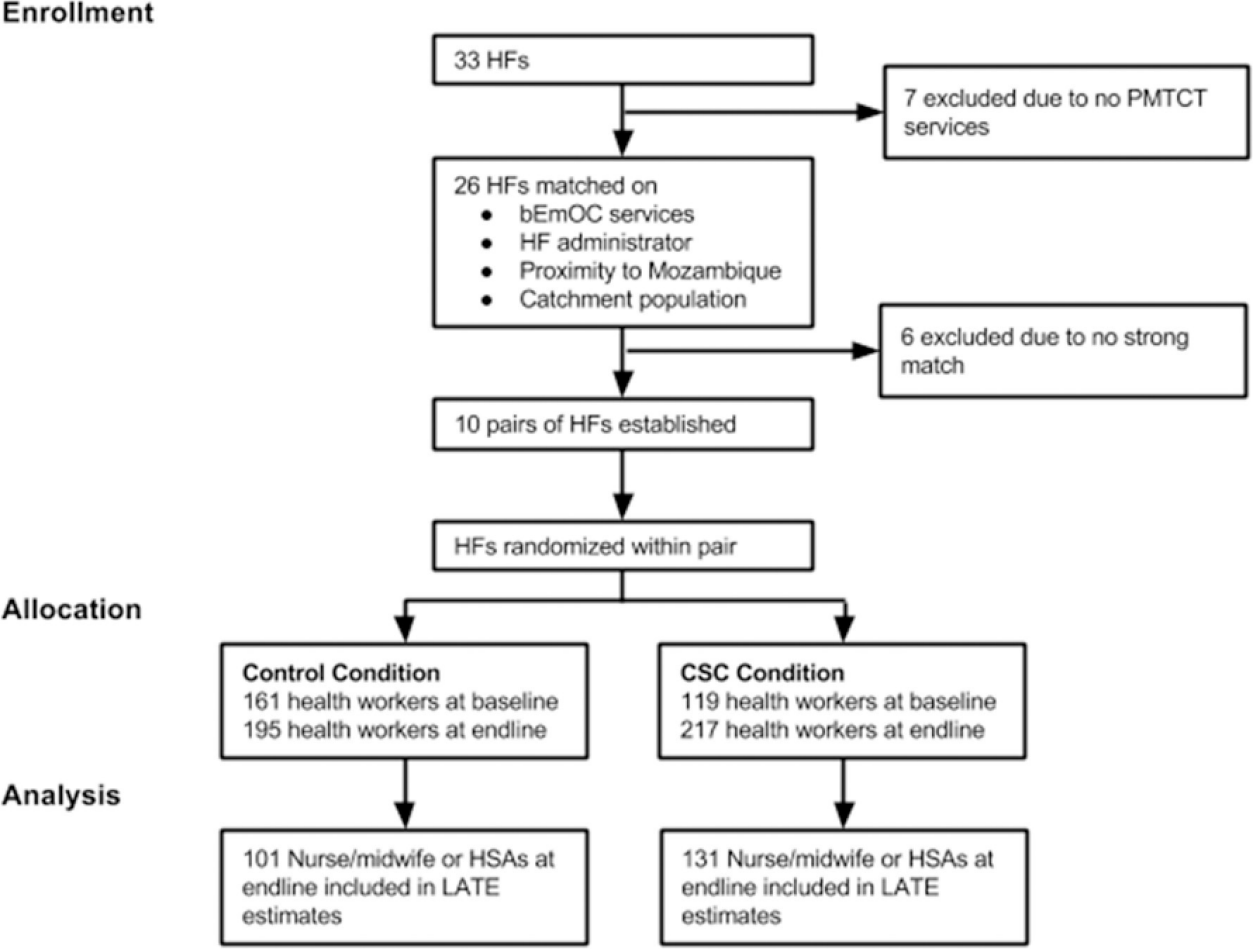
Randomization design flowchart. Footnote: HF: health facility; PMTCT: Prevention of Mother to Child Transmission of HIV; bEmOC: basic emergency obstetric care.

For our cluster-randomized design, we selected study sites based on health facility and surrounding catchment area, with 33 health facilities and surrounding catchment areas in the initial population. We excluded 13 of the 33 health facilities either because they did not provide PMTCT services or they did not have one or more of the required matching criteria. From the remaining health facilities, we created matched pairs based on four characteristics: presence of bEmOC services, facility administration (MOH or CHAM), proximity to the Mozambique border (which affects the population using the facility as well as the ability of health workers to provide services), and population size of the catchment area. This selection process resulted in ten matched pairs of health facilities. We randomly assigned one health facility from each pair to the treatment condition and the other to the control condition (Fig 3). The study was reviewed and approved by Malawi’s National Health Science Research Committee as a program evaluation.

### Data collection

We collected baseline data (n = 280) between November and December 2012 and endline data (n = 412) between November and December 2014. We attempted to survey to all health workers in the catchment areas of the twenty participating health facilities at both time points. An independent data collection team from the Centre for Development Management carried out the data collection. The health facility in-charge was the entry point at each health facility. Service providers were requested to participate in the interviews at least 48 hours in advance. A majority of the providers were interviewed at the health facility and the remaining in the health facility catchment areas. All participants provided consent prior to the start of the survey. The survey was quantitative and covered the following areas: participant identification, background and demographic characteristics, service responsibilities and provision, intervention exposure and participation, and a range of governance outcome-related topics (e.g., social cohesion). The questionnaire included a majority close-ended questions and only a small number of open-ended questions. All the data were self-reported. This paper focuses on the CSC’s impact on service responsibilities and provision.

### Measures

To assess service responsibilities, respondents indicated whether they had responsibility for each of the ten core health service domains: ANC, FP counseling, FP provision, HIV/PMTCT counseling, HIV testing, labor and delivery, postpartum follow-up, breastfeeding counseling, record keeping/tracking/monitoring, and supervision of other providers. Respondents were also asked if they performed record keeping functions in practice.

To evaluate service provision, respondents were asked questions related to working location and detailed questions on their provision of ANC and FP services. To assess working location, participants were asked the proportion of the working hours they spent at the health facility. ANC service provision questions for providers included the following: number of pregnant women and new mothers seen in the last month, proportion of pregnant women visited at home at least once during their pregnancy, number of times pregnant women were visited at home during their pregnancy, number of pregnant women counseled in the last month, and whether the most recent woman they provided services to received comprehensive ANC counseling. Comprehensive ANC counseling indicated that each of the six ANC topics had been discussed with all women seen in the past month: importance of getting check-ups during pregnancy; danger signs during pregnancy and childbirth; planning for delivery; getting tested for HIV; exclusive breastfeeding once the baby is born; and planning for contraception to avoid or delay another pregnancy after the baby is born.

FP service provision questions included the following: the number of women counseled about FP during the last month, details on who was provided FP services (i.e., the proportion of clients counseled on FP in the last month who were young women or adolescents as well as the average age, marital status and whether the client’s partner was present during the most recent FP consultation), and details on the most recent counseling session (i.e., whether counseling occurred in a private place, whether the woman was asked how long she wanted to wait before having a child/another child, whether HIV prevention was discussed, and whether comprehensive FP counseling was provided). Comprehensive FP counseling involved reporting all five recommended FP counseling practices during the most recent FP consultation: counseling the woman in a private place; asking how long she wanted to wait before having a child/another child; clarifying that it was her choice alone whether to use FP; discussing HIV prevention; and encouraging her to ask further questions.

Service provider self-reported ANC and FP service responsibility and provision items reported on here were developed to complement a more comprehensive set of questions around service provision and utilization included in the women’s survey and presented in Gullo et al. 2017 [17]. With the service provider survey we hoped to gain insights on the CSC’s impact on health worker responsibilities (e.g., did the CSC result in providers perceiving greater responsibilities?), working location (e.g., did the CSC result in more health workers providing services in the community?), clients served (e.g., did the CSC increase the number of adolescents receiving FP or men going with their partners for FP?) and additional details of FP and ANC service provision.

### Analysis

Respondents classified their role as one of four types of health workers. At endline, 2.4% of respondents identified as doctor/clinical officer, 10.9% as nurse/midwife, 29.4% as patient attendant, and 45.4% as HSAs. In addition, 11.9% of respondents selected ‘other’ with a write-in response. Analytic models presented here are limited to the 232 nurse/midwife and HSA respondents at endline. The other categories were not directly responsible for patient care: patient attendants primarily focus on patient support and facility upkeep (though many reported some patient care activities); doctors/clinical officers largely work in an administrative capacity and represent <3% of the endline sample. Write-in job titles were related to either administrative roles or to non-health care roles (e.g., security guards). Analysis focused on ANC and FP-related services. The other health services could not be evaluated because too few health workers (<70%) identified them as primary job responsibilities, there was a ceiling effect at baseline which left little room for change, or it was mostly the responsibility of just nurses/midwives of whom there were too few at endline (n = 45) to analyze separately.

We limited our analyses presented here to the endline-only data based on two considerations. First, health workers provided survey data anonymously which precluded the possibility of linking records from the same health workers across time. We expected that many of the same individuals were surveyed at both time points based on the 100% sampling plan, creating unaccountable dependencies between the baseline and endline data. Second, the total sample size increased unexpectedly from baseline to endline. Follow-up correspondence with project staff indicated that a greater awareness of the project at endline may have contributed to an increased participation rate; thus, the baseline and endline samples were nonequivalent.

Prior to analyzing treatment effects, treatment condition was regressed on demographic characteristics in individual logistic regression models that adjusted for catchment area clustering; the global null hypothesis (i.e., a model containing a single demographic variable is equivalent to an intercept-only model) was used to evaluate the possibility of non-equivalence across conditions. Treatment effects for all outcomes were estimated using both regression models to estimate intention to treat (ITT) and instrumental variable (IV) models to estimate local average treatment effects (LATE) [21]. In the ITT analysis, treatment was based on the condition to which the participant was assigned regardless of their participation in the CSC. The LATE estimates represent the treatment effect as if there had been perfect compliance with the random treatment assignment and is useful in adjusting for noncompliance in real-world data. Prior to fitting the models, we evaluated the extent of non-compliance and determined that the data contain both forms of non-compliance: treatment-area cases that did not participate in the CSC (34.4%) as well as control-area cases that did participate in the CSC (8.9%). All models were fit using the Stata 14.2 svy command in which catchment area was the primary sampling unit (i.e., cluster); standard errors were thus estimated using robust variance estimates. All models included the following covariates: age, religion (Catholic v. not Catholic), sex, ethnicity (Ngoni v. not Ngoni), location where the health worker lives (at the facility, in the community, or does not live in the community), electricity in home, and the time required to reach a main road (less than 30 minutes, 30–59 minutes, 1–2 hours, more than 2 hours).

## Results

### Sample characteristics

Socio-demographic and household characteristics of HSAs and nurses/midwives are outlined in Table 1. A majority of the nurses/midwives are 40 years or older and are mostly female. However, the majority is also of non-Ngoni ethnicity and live in the facilities where they work with a metal roof and electricity. The majority of nurses/midwives have been in their current position for more than 2 years. The majority of HSAs are 31–40 years-old, mostly male, and of Ngoni ethnicity. They tend to live in one of the communities where they work, in homes with a metal roof but no electricity. Like the nurses/midwives, nearly all the HSAs have been in their current position for more than 2 years. The only significant difference in socio-demographic and household characteristics of HSAs and nurses/midwives between the treatment and control areas was that fewer HSAs and nurses/midwives in the treatment areas lived in the communities in which they worked (F[2, 18] = 3.94, p =. 038). The similarities between the treatment and control areas suggest that our cluster randomization process worked well in generating equivalence in individuals across conditions though, while not accounting for significant effects, nurses/midwives appeared to exhibit greater variability than HSAs.

**Table 1:** Selected socio-demographic and household characteristics of health workers: Endline, 2014.

Characteristics	Control		Treatment	
	Health Surveillance Assistant (n = 81)	Nurse/ Midwife (n = 20)	Health Surveillance Assistant (n = 106)	Nurse/ Midwife (n = 25)
**Age**				
20–30	16.0%	40.0%	17.9%	44.0%
31–40	54.3%	5.0%	56.6%	24.0%
41–50	25.9%	15.0%	17.0%	12.0%
51+	3.7%	40.0%	8.5%	20.0%
**Religion**				
Catholic	19.8%	40.0%	20.8%	64.0%
Presbyterian	30.9%	5.0%	28.3%	12.0%
Other Christian	39.5%	45.0%	42.5%	24.0%
Other	9.9%	10.0%	8.5%	0.0%
**Sex**				
Male	65.4%	30.0%	54.7%	40.0%
Female	34.6%	70.0%	45.3%	60.0%
**Ethnicity**				
Other	29.6%	65.0%	41.5%	80.0%
Ngoni	70.4%	35.0%	58.5%	20.0%
**Do you live in one of the communities where you work?**^**a**^				
Yes, at the institution/facility	9.9%	95.0%	8.5%	88.0%
Yes, in the community	81.5%	0.0%	67.9%	12.0%
No	8.6%	5.0%	23.6%	0.0%
**Metal roof on home**				
Roof on home is not metal	4.9%	0.0%	5.7%	0.0%
Metal roof on home	95.1%	100.0%	94.3%	100.0%
**Electricity in home**				
No electricity, solar power or generator in home	69.1%	20.0%	71.7%	8.0%
Electricity, solar power or generator in home	30.9%	80.0%	28.3%	92.0%
**Four or more years since graduation**				
Four years or less since graduation	3.7%	35.0%	2.8%	40.0%
More than four years since graduation	96.3%	65.0%	97.2%	60.0%
**Two or more years in position**				
Two years or less in position	0.0%	30.0%	1.9%	28.0%
More than two years in position	100.0%	70.0%	98.1%	72.0%
**How far are you from a place to get basic supplies?**				
No time (basic supplies in town)	2.5%	0.0%	0.9%	0.0%
Less than 30 minutes	49.4%	45.0%	38.7%	28.0%
30–59 minutes	17.3%	15.0%	15.1%	36.0%
1–2 hours	19.8%	25.0%	26.4%	12.0%
More than 2 hours	11.1%	15.0%	18.9%	24.0%
**How far are you from a main road?**				
No time (main road in town)	3.7%	10.0%	3.8%	4.0%
Less than 30 minutes	50.6%	50.0%	42.5%	56.0%
30–59 minutes	13.6%	15.0%	16.0%	16.0%
1–2 hours	24.7%	20.0%	23.6%	12.0%
More than 2 hours	7.4%	5.0%	14.2%	12.0%

^**a**^ Significant difference between HSAs and nurses/midwives in treatment areas vs. control areas (F[2, 18] = 3.94, p =. 038)

### Service responsibilities

HSA and nurse/midwife reported responsibilities across both treatment and control areas are outlined in Table 2. Health workers self-report being responsible for a wide range of sexual, reproductive, and maternal health services. HSAs nearly universally report being responsible for counseling about FP and breastfeeding across both treatment and control areas; approximately three-quarters report being responsible for providing FP and ANC, for HIV/PMTCT counseling, and for monitoring pregnant and postpartum women. Less than half of HSAs report being responsible for postpartum follow-up; however, approximately one-third report some supervisory responsibility of other providers.

**Table 2:** Health worker-reported responsibilities across both treatment and control areas, by type of health worker: Endline, 2014.

Category	Variable	Health Surveillance Assistant	Nurse/ Midwife
**Core health service responsibilities** (% of health workers that indicated that they are responsible for service)	**Antenatal care**	75.9%	97.8%
	**Family planning counseling**	91.4%	77.8%
	**Family planning provision**	74.9%	55.6%
	**HIV/PMTCT counseling**	71.1%	80.0%
	**HIV testing**	19.8%	55.6%
	**Labor and delivery**	1.6%	97.8%
	**Postpartum follow-up**	42.2%	93.3%
	**Breastfeeding counseling**	93.0%	100.0%
	**Record keeping/ tracking/ monitoring of pregnant & postpartum women**	72.7%	82.2%
	**Supervision of other providers**	34.2%	88.9%
**Record keeping**	**Do you record how any pregnant women or new mothers you see each month?**	77.9%	94.4%

A large majority of nurses/midwives across both treatment and control areas report being responsible for all the sexual, reproductive, and maternal health services assessed. Providing FP and HIV testing are the services for which the fewest number of nurses/midwives claimed responsibility, yet more than half did provide these services. Interestingly, 75% of HSAs across both treatment and control areas report being responsible for FP provision while 56% of nurses/midwives report having this responsibility. In addition, a little more than 77% of HSAs and 94% of nurses/midwives report recording the number of pregnant women or new mothers they see each month.

### Service provision

HSA and nurse/midwife reported service provision across both treatment and control areas is outlined is Table 3. A little over 40% of HSAs and 97% of nurses/midwives from both treatment and control areas report spending half or more of their working hours at the health facility. Both HSAs and nurses/midwives report seeing a large number of pregnant women as well as counseling a large number for ANC during the past month. Fifty-five percent of HSAs report visiting all pregnant women in their catchment area at home during the last month whereas only 12% of nurses/midwives do, which likely reflects the HSAs’ role in the community. Eighty-seven percent of HSAs and 30% of nurses/midwives report visiting pregnant women at home in their catchment area 2 or more times during their pregnancy. A majority of HSAs and nurses/midwives report asking pregnant women if they plan to avoid or delay another pregnancy after their baby is born. A little over half of HSAs (51.8%) and a majority of nurses/midwives (75.0%) across both treatment and control areas report providing comprehensive ANC counseling to all of their clients in the past month.

**Table 3:** Distribution of health worker-reported service provision outcomes across both treatment and control areas by type of health worker.

Variable	Health Surveillance Assistant	Nurse/ Midwife
**Working location**		
**Proportion of your working hours did you work at the health facility?**		
None of my time (0%)	2.1%	0.0%
Only a little of my time (<25%)	36.4%	0.0%
Less than half of my time (25–49%)	20.9%	2.2%
At least half of my time (50–74%)	26.2%	6.7%
Most of my time (75% or more)	12.8%	22.2%
All of my time (100%)	1.6%	68.9%
**Antenatal care (ANC)**		
**How many pregnant women or new mothers did you see in the last month?**		
0	11.7%	5.6%
1–10	58.5%	5.6%
11–50	24.0%	38.9%
51+	5.8%	50.0%
**Pregnant women visited at home at least once during their pregnancy?**		
None	7.9%	76.5%
A few	17.1%	11.8%
Half	7.9%	0.0%
Most	11.8%	0.0%
All	55.3%	11.8%
**Number of times visited pregnant women in catchment area at home during their pregnancy?**		
Never	2.6%	70.6%
One time only	10.5%	0.0%
2 or 3 times	58.6%	11.8%
4 times	12.5%	5.9%
More than 4 times	15.8%	11.8%
**Last month, how many pregnant women did you counsel?**		
0	10.3%	2.2%
1–10	50.5%	11.1%
11–50	28.3%	33.3%
51+	10.9%	53.3%
**Planning for contraception to avoid or delay another pregnancy after the baby is born?**		
None	6.5%	2.3%
A few	11.9%	0.0%
Half	6.0%	9.1%
Most	5.4%	4.5%
All	70.2%	84.1%
**Comprehensive ANC counseling**	51.8%	75.0%
**Family Planning (FP)**		
**During the last month, how many women did you counsel about family planning?**		
0	5.9%	11.1%
1–10	20.5%	11.1%
11–50	40.0%	37.8%
51+	33.5%	40.0%
**Comprehensive FP counseling**	56.8%	67.5%
**Were you able to counsel her in a private place where no one could overhear your conversation?**	88.6%	90.0%
**Did you ask her how long she wanted to wait before having a child/another child?**	74.4%	77.5%
**Did you talk to her about HIV prevention?**	85.8%	95.0%
**Of these women you counseled during the last month, about how many were young women or adolescents?**		
None	23.3%	10.0%
A few	45.5%	27.5%
Half	11.4%	30.0%
Most	15.3%	27.5%
All	4.5%	5.0%
**About how old was the last woman you counseled about family planning?**		
15–19	21.6%	24.3%
20–24	29.2%	40.5%
25–29	28.1%	13.5%
30–34	12.9%	8.1%
35–45	8.2%	13.5%
**Thinking about the last time you provided family planning services to a woman, was she currently married?**	89.2%	72.5%
**Thinking about the last time you provided family planning services to a woman; did she come with her partner?**	11.9%	15.0%

Both HSAs and nurses/midwives in treatment and control areas report counseling a large number of women on FP each month. Just over 31% of HSAs and 62.5% of nurses/midwives report that half or more of the clients they saw in the last month were young women or adolescents. Furthermore, 21.6% of HSAs and 24.3% of nurses/midwives reported the last client they counseled on FP was 15–19 years old. A majority of both HSAs and nurses/midwives reported the last woman they provided FP services to was married, however, a minority of both reported the last woman they provided services to brought her partner. A majority of both HSAs and nurses/midwives reported that the last woman they counseled was counseled in a private place, was asked how long she wanted to wait before having a child/another child, and was counseled on HIV prevention. Over half of HSAs (56.8%) and 67.5% of nurses/midwives in both treatment and control areas report providing comprehensive FP counseling.

### ITT and LATE

The ITT and LATE estimates of the CSC’s effect are outlined in Table 4. Both ITT and LATE estimates indicate that at endline a significantly greater proportion of HSAs and nurses/midwives in the treatment areas report being responsible for providing ANC than those in the control areas (p = .004 and p = .005, respectively). However, both ITT and LATE estimates indicate HSA and nurse/midwife reported responsibility for HIV testing was lower in treatment than in control areas (p = .041 and p = .038, respectively). Both ITT and LATE estimates indicate that a marginally significant (i.e., p < .10) proportion of HSAs and nurses/midwives in the treatment areas compared to those in the control areas reported visiting pregnant women in their catchment areas at least once during their pregnancy. ITT and LATE estimates both indicate a significantly greater proportion of HSAs and nurses/midwives in the treatment areas reported providing comprehensive ANC (p = .062 and p = .011, respectively) than in the control areas. Similarly, a greater proportion of HSAs in the treatment areas also reported recording the number of pregnant and postpartum women that they see each month than HSAs in the control areas (p < .001). ITT and LATE estimates of the CSC’s effect indicate that women receiving FP counseling are younger in the treatment areas than in the control areas (p = .026 and p = .023, respectively).

**Table 4:** CSC impact on health worker-reported responsibilities and service provision outcomes among nurses/midwives and HSAs by treatment vs. control areas: Local average treatment effect (LATE) estimates, endline, 2014^1^.

Variable	Outcome	ITT Models				LATE Models			
		ITT	95% CI	*t*	*p*	LATE	95% CI	*t*	*p*
**Service responsibilities**									
**Responsibility for core healthcare services**	Antenatal care	0.10	0.04–0.17	3.29	0.004	0.18	0.06–0.31	3.17	.005
	Family planning provision	-0.07	-0.22–0.09	-0.94	0.361	-0.12	-0.41–0.16	-0.91	.374
	HIV/PMTCT counseling	0.04	-0.09–0.17	0.63	0.539	0.07	-0.16–0.29	0.63	.534
	HIV testing	-0.08	-0.15 - -0.00	-2.19	0.041	-0.14	-0.26 - -0.01	-2.23	.038
	Labor and delivery	-0.00	-0.06–0.05	-0.12	0.909	-0.01	-0.11–0.10	-0.12	.908
	Postpartum follow-up	0.15	-0.02–0.32	1.80	0.087	0.26	-0.04–0.57	1.80	.088
	Record keeping/ tracking/ monitoring of pregnant & postpartum women	0.09	-0.02–0.20	1.67	0.111	0.16	-0.03–0.34	1.75	.096
	Supervision of other providers	-0.05	-0.20–0.11	-0.63	0.538	-0.08	-0.37–0.20	-0.62	.546
	Proportion of your working hours did you work in the community?	-0.01	-0.47–0.45	-0.05	0.958	-0.02	-0.84–0.80	-0.05	.958
**Record keeping**	Do you record how any pregnant women or new mothers you see each month?^2^	0.26	0.17–0.35	6.07	< .001	0.44	0.31–0.58	7.03	< .001
**Service provision**									
**Antenatal care (ANC)**	Pregnant women visited at home at least once during their pregnancy? ^2^	0.53	-0.03–1.09	1.98	0.062	0.93	-0.10–1.97	1.89	.075
	Number of times visited pregnant women in catchment area at home during their pregnancy? ^2^	0.02	-0.33–0.38	0.13	0.899	0.04	-0.59–0.66	0.13	.899
	Comprehensive ANC counseling	0.18	0.05–0.30	2.95	0.008	0.31	0.08–0.53	2.80	.011
**Family planning (FP)**	Comprehensive FP counseling	0.02	-0.13–0.17	0.33	0.749	0.04	-0.23–0.31	0.33	.747
	Of these women you counseled during the last month, about how many were young women or adolescents?	0.15	-0.12–0.41	1.17	0.257	0.26	-0.21–0.73	1.15	.264
	About how old was the last woman you counseled about family planning?	-1.40	-2.61 - -0.18	-2.41	0.026	-2.53	-4.69 - -0.38	-2.46	.023
	Thinking about the last time you provided family planning services to a woman, was she currently married?	0.05	-0.06–0.15	0.87	0.395	0.08	-0.11–0.27	0.88	.388
	Thinking about the last time you provided family planning services to a woman, did she come with her partner?	0.04	-0.08–0.16	0.65	0.522	0.07	-0.15–0.28	0.65	.521

^1^ LATE estimates represent percentage difference for binary outcomes and mean difference for continuous outcomes. All LATE models contained CSC participation instrumented on treatment assignment, and all ITT and LATE models contained the following covariates: age, religion, sex, ethnicity, location where health worker lives, electricity in home, and the time required to reach a main road.

^2^Nurses/midwives excluded from model.

## Discussion

In order to more fully understand the CSC’s impact on health worker responsibilities and service provision we carried out a quantitative survey with health workers in Malawi. We found that significantly more health workers in the CSC intervention communities compared to the control communities self-report the following at endline: responsibility for ANC, comprehensive counseling during ANC, recording of the number of pregnant and postpartum women seen each month, and the average age of their last FP client served was younger. In addition, marginally significantly more health workers in treatment versus control areas report visiting women at their home at least once during their pregnancy. To our knowledge, this is the first quantitative survey of health workers as part of collaborative social accountability approach evaluation efforts. Ensuring health workers and women both had voice in the CSC’s evaluation was important given the approach’s aim is to empower both, recognizing that quality is the product of a linked chain [4,5,6,7].

Significantly more health workers in the CSC intervention communities than control reported responsibility for ANC. Score Card data may be able to shed some light on this finding. Through the CSC process, providers and community members identified that availability and accessibility of maternal and newborn health (MNH) and FP information and services were barriers to reproductive health service utilization. In digging into these issues to come up with solutions, it was discovered that not all HSAs or community members knew that MNH care is a responsibility of HSAs. Therefore, actions were taken to train and support HSAs in delivering MNH. Analysis of the Score Card data showed that providers and community members reported improvements in both availability and accessibility of MNH and FP information and health services, with the availability and accessibility of information improving significantly [17]. A study in Ethiopia found role ambiguity to be a major stressor for CHWs [3]. The CSC aims to surface and overcome this type of provider-side stressor and obstacle alongside community-side challenges in efforts to improve service utilization and delivery.

Marginally significantly more health workers in treatment versus control areas report visiting women at their home at least once during their pregnancy. These findings are important because they align with the Malawi MOH expectations that HSAs should provide their services through door-to-door visits [22]. They also converge with our evaluation findings from the women’s survey data showing that the CSC increased the proportion of women who reported receiving a home visit during pregnancy [17]. The Score Card data and actions resulting from the CSC process shed light on this finding. The CSC process helped to facilitate support for HSAs from both the health system and community (e.g., capacity building from the district government, improvements in supervision, improvements in the relationship with the community, and community mobilization of resources to build HSA houses and clinics) [17]. While the CSC increased women receiving home visits during pregnancy, it also supported HSAs to do so. As George 2003 [10] suggests, accountability approaches that support interactions between communities and services can benefit both sides.

The CSC was also associated with more comprehensive counseling during ANC visits and increased monitoring and record-keeping. After years of primarily encouraging pregnant women in lower-income countries to obtain ANC and increase the number of visits they receive during pregnancy, recent efforts have focused on how to improve and measure the quality of those ANC visits [23]. Though comprehensive counseling is not the only indicator of ANC quality, it is an important indicator—especially in a low-resource setting like Malawi where ANC serves to educate women and build a relationship between her, her family, and the health system. In addition, most HSAs and nurses/midwives in both the treatment and control areas reported being responsible for monitoring and tracking pregnant women, but health workers in the treatment areas reported higher levels of actually doing so. Being able to track and monitor pregnant women in their catchment area is an important step in being able to follow-up with them, make referrals, and deliver quality care. Record-keeping by frontline health workers also helps from a health systems perspective because it provides a mechanism to monitor demand and capacity, and then to make adjustments as necessary. For this reason, data tracking by the health workforce is a key component of the WHO’s Global Strategy on Human Resources for Health: Workforce 2030 initiative [24]. These improvements in counseling and tracking converge with previous findings from our evaluation that the CSC significantly improved providers commitment as well as improved women’s satisfaction with services in the treatment areas [17].

The average age of the last FP client served was younger in the intervention communities versus control. The association we found between the treatment areas and slightly younger age for the last woman provided FP counseling may indicate a shift in the characteristics of women accessing FP and/or for whom providers feel that FP counseling is appropriate. Through the CSC process, increasing involvement of youth in reproductive health was an area identified by service providers and community members as important for accelerating reproductive health progress. In addition, it was an area that community members and service providers perceived improved significantly due to the CSC process [17]. Evidence suggests that provider bias poses a barrier to adolescents accessing and utilizing FP information and services [25,26,27,28]. The CSC may make health workers more amenable to providing FP counseling and services to adolescents as their knowledge of patients’ rights increases and as they see increased community investment in and commitment to reproductive health for youth. This is in line with research that suggests that social accountability approaches that emphasize dialogue and negotiation can help to mitigate social biases or prejudices among health professionals and improve their receptivity to clients’ needs [10]. Increasing adolescent and young women’s access to FP is a key priority of the Family Planning 2020 Initiative to which Malawi has committed itself [29]. Social accountability approaches that attack and overcome the demand and supply-side issues may hold promise in advancing towards this goal.

While the CSC did improve some aspects of service provision, there are some notable areas where the CSC appeared to have a negative effect or no effect at all. Interestingly, the CSC was associated with lower health worker-reported responsibility for HIV testing. We do not know how this occurred; however, it could be a result of HSA role and responsibility clarification that occurred as a result of the CSC process. Self-reported job responsibilities and service provision tend to match with MOH expectations for HSAs [22] across both the treatment and control areas, however, there are two notable exceptions. First is the 40% of HSAs who, even at endline, report spending half or more of their working hours in a health facility instead of in the community. The Malawi MOH established the HSA role as a community health worker whose main responsibilities involve visiting families in their homes to deliver promotive and preventive health services [30]. It appears that a large percentage of HSAs do not follow this community-based guideline. In addition, less than half of HSAs report being responsible for postpartum follow-up despite it being an official part of their job responsibilities and critical to accelerating maternal health efforts in the country. Perhaps these issues require greater focus in the CSC process in order for providers and community members to identify the obstacles and solutions to remedy these issues. Or perhaps the reason for the CSC not positively affecting these issues is that they require central level health system responsiveness, which the CSC and similar CVA have had limited success in garnering [13,16,17,18]. Despite finding that many HSAs report spending a significant proportion of their working hours in a health facility and a lower than expected percentage report responsibility for postpartum care, we found the CSC intervention significantly increased HSA visits to women during pregnancy and during the postnatal period compared to control [17].

### Limitations

Several limitations may have affected our ability to identify significant differences in the treatment areas. First, we elected to accelerate the endline data collection by a full year because other organizations began planning maternal child health-related interventions and services in the control areas which would have contaminated our evaluation design. However, systemic changes facilitated by the CSC process may require a longer time period to operate in order to observe anticipated effects on service delivery outcomes. Second, we were unable to use the baseline data as planned to assess the impact of the intervention on service delivery outcomes over time. This limited us to estimating a treatment effect via LATE models at endline-only, a method which cannot rule out group differences in existence prior to intervention; thus, effects should be interpreted conservatively. Third, while the models adjusted for non-independence of observations attributable to catchment areas and a variety of person-level characteristics, we did not possess catchment-level characteristics that could be used to estimate contextual effects. Fourth, as the survey is with health workers about their responsibilities and service provision, it is subject to recall bias and social desirability bias. As the analysis was conducted after the intervention, there is some possibility that healthcare providers exposed to the intervention are aware of what is expected which could increase a positive response bias. There were hopes to use facility records to triangulate the findings; however, the high number of missing records and poor quality of recordkeeping made them unusable. Fifth, while carrying out a quantitative survey with health workers allowed a much broader sample, we were unable to gain the depth in understanding that a qualitative approach would have allowed. Sixth, the ‘Cashgate’ scandal froze donor funds to Malawi for a while during the intervention period [31] and strained the country’s public services including the health system. Finally, randomization was conducted at the health center catchment area and not the health worker level; nevertheless, health workers only differed in the proportions living in the community across treatment conditions.

## Conclusions

The CSC aims to empower health workers to collaborate with the community and rest of the health system to identify and overcome the diverse and context-specific range of performance barriers they face. In doing so, it supports them to demand and ensure quality care for themselves from the health system so they can, in turn, deliver quality services to clients. Here we present the CSC’s impact on health workers’ self-reported service responsibilities and provision in Malawi. These results, taken with the women’s survey and Score Card data results [17], suggest the CSC may hold promise at improving service provision. While there is increasing evidence that collaborative social accountability approaches like the CSC are effective means to improve reproductive health-related service provision and outcomes in low-resource settings, additional research is needed. Research, including qualitative and realist evaluations, is needed to unpack the black box of how these approaches work as well as understand stakeholders’ (including frontline service providers’) experiences of and satisfaction with these approaches.

## Supporting information

S1 File Data. (XLS)
